# Genetic Diversity, Biofilm Formation, and Antibiotic Resistance of *Pseudomonas aeruginosa* Isolated from Cow, Camel, and Mare with Clinical Endometritis

**DOI:** 10.3390/vetsci9050239

**Published:** 2022-05-16

**Authors:** Samy F. Mahmoud, Mahmoud Fayez, Ayman A. Swelum, Amal S. Alswat, Mohamed Alkafafy, Othman M. Alzahrani, Saleem J. Alsunaini, Ahmed Almuslem, Abdulaziz S. Al Amer, Shaymaa Yusuf

**Affiliations:** 1 Department of Biotechnology, College of Science, Taif University, Taif 21944, Saudi Arabia; s.farouk@tu.edu.sa (S.F.M.); a.alswat@tu.edu.sa (A.S.A.); m.kafafy@tu.edu.sa (M.A.); 2 Al-Ahsa Veterinary Diagnostic Laboratory, Ministry of Environment, Water and Agriculture, Al-Ahsa 31982, Saudi Arabia; sslam0002@gmail.com (S.J.A.); aaa4481@hotmail.com (A.A.); dr-aziz18@hotmail.com (A.S.A.A.); 3 Department of Bacteriology, Veterinary Serum and Vaccine Research Institute, Ministry of Agriculture, Cairo 12618, Egypt; 4 Department of Theriogenology, Faculty of Veterinary Medicine, Zagazig University, Zagazig 44511, Egypt; aymanswelum@zu.edu.eg; 5 Department of Biology, College of Science, Taif University, Taif 21944, Saudi Arabia; o.alzahran@tu.edu.sa; 6 Department of Microbiology, Faculty of Veterinary Medicine, Assiut University, Assiut 71515, Egypt; shaymaayusuf@yahoo.com

**Keywords:** antimicrobial resistance, *Pseudomonas aeruginosa*, carbapenem resistance, MLST, virulence genes, multidrug resistance, antimicrobial-resistance genes

## Abstract

*Pseudomonas aeruginosa* is a ubiquitous opportunistic bacterium that causes diseases in animals and humans. This study aimed to investigate the genetic diversity, antimicrobial resistance, biofilm formation, and virulence and antibiotic resistance genes of *P. aeruginosa* isolated from the uterus of cow, camel, and mare with clinical endometritis and their drinking water. Among the 180 uterine swabs and 90 drinking water samples analysed, 54 (20%) *P. aeruginosa* isolates were recovered. Isolates were identified biochemically to the genus level by the automated Vitek 2 system and genetically by the amplification of the *gyrB* gene and the sequencing of the *16S rRNA* gene. Multilocus sequence typing identified ten different sequence types for the *P. aeruginosa* isolates. The identification of ST2012 was significantly (*p* ≤ 0.05) higher than that of ST296, ST308, ST111, and ST241. The isolates exhibited significantly (*p* ≤ 0.05) increased resistance to piperacillin (77.8%), ciprofloxacin (59.3%), gentamicin (50%), and ceftazidime (38.9%). Eight (14.8%) isolates showed resistance to imipenem; however, none of the isolates showed resistance to colistin. Multidrug resistance (MDR) was observed in 24 isolates (44.4%) with a multiple antibiotic resistance index ranging from 0.44 to 0.77. MDR was identified in 30 (33.3%) isolates. Furthermore, 38.8% and 9.2% of the isolates exhibited a positive extended-spectrum-β-lactamase (ESBL) and metallo-β-lactamase (MBL) phenotype, respectively. The most prevalent β-lactamase encoding genes were *bla_TEM_* and *bla_CTX-M_*, however, the *bla_IPM_* gene was not detected in any of the isolates. Biofilm formation was observed in 49 (90.7%) isolates classified as: 11.1% weak biofilm producers; 38.9% moderate biofilm producers; 40.7% strong biofilm producers. A positive correlation was observed between the MAR index and biofilm formation. In conclusion, the results highlighted that farm animals with clinical endometritis could act as a reservoir for MDR and virulent *P. aeruginosa*. The emergence of ESBLs and MBLs producing *P. aeruginosa* in different farm animals is a public health concern. Therefore, surveillance programs to monitor and control MDR *P. aeruginosa* in animals are required.

## 1. Introduction

*Pseudomonas aeruginosa* is an opportunistic Gram-negative motile bacterium. Because of its remarkable metabolic plasticity, *P. aeruginosa* is ubiquitously distributed in many ecological niches including soil, water, hospital environments, and animal ecosystems [1,2,3,4]. *P. aeruginosa* has been linked to a wide range of life-threatening infections, particularly in immunocompromised people. It has been of significant relevance since it is the primary cause of morbidity and death in people with cystic fibrosis and is one of the most common nosocomial bacteria infecting hospitalised people [5,6]. The impact of *P. aeruginosa* is not only restricted on human health but also extends to animal production [7].

Animal production is influenced by reproductive diseases that reduce fertility [8,9]. Endometritis and metritis are the most prevalent reproductive disease of cattle [10], camel [11], and mare [12], causing a reduction in total milk yield [11,13,14]. Bacterial infection is the common cause of uterine inflammation, which can occur during or shortly after parturition, coitus, or artificial insemination. Various pathogenic bacteria have been associated with animal reproductive tract infection including *Streptococcus* spp., *Staphylococcus* spp., *Escherichia coli*, *Clostridium* spp., *Fusobacterium necrophorum*, *P. aeruginosa*, *Bacillus* spp., and *Corynebacterium* spp. [15,16,17]. *P. aeruginosa* causes reproductive tract infection in cattle, equine, and camel [12,15,18], in addition to mastitis [19] and cystitis [20] in livestock. In companion animals, *P. aeruginosa* causes urinary tract infection [21], otitis [22], and pyoderma [23].

Animal reproductive tract infection is commonly treated with an intrauterine infusion or the systemic administration of antibiotics. However, the improper use of antimicrobials leads to antibiotic resistance, which endangers both human and animal health [9]. *P. aeruginosa* currently displays resistance to various antibiotics including β-lactams, quinolones, and aminoglycosides [24]. The World Health Organisation has listed *P. aeruginosa* as a critical pathogen in the research and development of new antibiotics [25].

Antimicrobial resistance in *P. aeruginosa* is attributed to three major types of mechanisms: intrinsic, acquired, and adaptive resistance. The intrinsic antimicrobial resistance of *P. aeruginosa* is due to the expression of efflux pumps, low outer membrane permeability, and the production of antibiotic-inactivating enzymes [26]. The acquired resistance can be established by the horizontal transfer of variable resistance genes including β-lactamases, metallo-β-lactamases, and aminoglycoside resistance genes [27,28,29,30]. However, the adaptive resistance of *P. aeruginosa* involves the formation of biofilm [31].

Biofilms are dense bacterial populations adhered to a solid surface and encased in an exopolysaccharide matrix. Bacterial cells grown in biofilms are more resistant to antimicrobial agents and phagocytosis than planktonic bacteria [32]. Biofilm formation in *P. aeruginosa* is mainly regulated by the quorum-sensing system (QS), a cell-to-cell signalling that regulates gene expression in response to changes in cell population density [33]. Three QS systems, LasI-LasR, RhlI-RhlR, and PQS-MvfR, were identified in *P. aeruginosa* to all generate autoinducers, which diffuse into bacterial and host cells, leading to transcriptional regulation that promotes bacterial survival and reduces the immune response to infection [34,35,36,37,38].

Drinking water quality and the drinking water system significantly impact the livestock’s general health and performance [39]. Waterborne pathogens can cause a potential risk for human and animal health [40]. The number of microorganisms in water can be increased when conditions are favourable or when they possess the ability to form biofilm. *E. coli*, *Enterococcus* spp., *Pseudomonas* spp., and *Salmonella* spp. are the most frequent biofilm-producing bacteria isolated from animal drinking water [41,42,43].

Several molecular typing methods have been used to type *P. aeruginosa* strains such as PCR-based fingerprinting [44,45], pulsed-field gel electrophoresis [46], and the multilocus sequence typing (MLST) scheme, which are based on differences in the sequences of seven housekeeping genes [37].

In the last few decades, the resistance of *P. aeruginosa* to antimicrobial agents has grown in Saudi Arabia with an increasing prevalence of extended-spectrum β-lactamase and metallo-β-lactamase producers in both the nosocomial and community [47,48,49,50,51,52]. However, the published literature describing the prevalence of *P. aeruginosa* in animals remains scarce.

The One Health approach has gained worldwide recognition as a valuable way to address critical public health issues including the problem of antimicrobial drug resistance at the human–animal–environment interface. Therefore, the main goals of this study were: (1) To investigate the prevalence of *P. aeruginosa* in different animal species with endometritis and their environment in the eastern province, Saudi Arabia; (2) To analyse the genetic heterogeneity of the isolates by MLST; (3) To determine the phenotypic antibiotic susceptibility, biofilm formation profiles, the molecular identification of virulence, and the antibiotic resistance genes in the isolates.

## 2. Materials and Methods

### 2.1. Animals

The study was conducted from May 2020 to February 2021 in the Eastern Province, Saudi Arabia. A total of 180 pluriparus animals (cow, *n* = 70, aged 3–7 years; camel, *n* = 60, aged 7–13 years; mares, *n* = 50, aged 5–9 years) that belonged to 90 small farms (30 farms/animal species) that had suffered from repeat breeder syndrome were selected. All animals exhibited clinical endometritis based on the clinical investigation criteria described by Refaat et al. and LeBlanc et al. [53,54]. The production system is seminomadic for camels and intensive for cow and mare. None of the animals received antibiotic treatment before sampling.

### 2.2. Samples

Before sampling, the external genitalia were cleaned with warm water and soap, disinfected with 0.1% iodopovidone, and dried with a clean towel. The uterine swabs were collected by passing a sterile double-guarded uterine culture swab (Minitüb, Tiefenbach, Germany) through the cervix, guided by a gloved arm, rotated to the right and left over the uterine wall. Afterward, the swab was retracted into the protecting tube and removed from the animal. Swabs were then broken into sterile 2 mL microfuge tubes containing 1% peptone water (Oxoid, Basingstoke, UK).

A total of 90 drinking water samples were collected in sterile plastic bottles from cattle farms (*n* = 30), camel herds (*n* = 30), and equine stables (*n* = 30). All samples were labelled and transported to the lab in the icebox for bacteriological examination.

### 2.3. Bacterial Isolation

The microfuge tubes and water samples were gently vortexed and an aliquot of 10 μL was streaked onto pseudomonas cetrimide agar (PCA) and brain heart infusion (BHI) agar (Oxoid, Basingstoke, UK) and incubated aerobically at 37 °C for 24 h. Growths were examined for colony morphology, Gram staining, and the oxidase test. Gram-negative isolates that were oxidase-positive were subjected to further biochemical identification by the Vitek 2 compact system using GN identification cards (BioMérieux, Marcy L’Etoile, France).

### 2.4. Molecular Conformation of the Isolates

For the isolation and purification of the total genomic DNA, the QIAamp DNA Mini-kit (Qiagen SA, Courtaboeuf, Les Ulis, France) was used according to the manufacturer’s instructions. Purified DNA was amplified by real-time PCR for the detection of *gyrB* genes, according to Hosu et al. [55]. For the amplification of the 16S rRNA gene, the primers 27F (5′-AGAGTTTGATCCTGGCTCAG-3′) and 1492R (5′-TACGGYTACCTTGTTACGACTT-3′) were used according to Weisburg et al. [56]. The PCR products were purified (QIAquick PCR Purification Kit, Qiagen, Les Ulis, France) and sequenced using an ABI 3500 Genetic analyser (Applied Biosystems, Bedford, MA, USA). Sequences were subjected to analysis through the National Centre for Biological Information (NCBI) Basic Local Alignment Search Tool (https://blast.ncbi.nlm.nih.gov/Blast.cgi, accessed on 10 January 2022).

### 2.5. Molecular Typing

To determine the sequence types (STs) among the isolates, seven house-keeping genes (*acsA, aroE, guaA, mutL, nuoD, ppsA*, and *trpE*) were amplified and sequenced according to the methods of Curran et al. [37]. STs were obtained by comparing the sequence with the reference *P. aeruginosa* MLST database (https://pubmlst.org/organisms/pseudomonas-aeruginosa) (accessed on 10 January 2022). Alignment and phylogenetic reconstructions for the concatenated sequences of the seven housekeeping genes were performed using MEGA (version 11) software.

### 2.6. Antimicrobial Susceptibility Testing

Nine antimicrobials that are commonly used to treat *P. aeruginosa* were selected for *P. aeruginosa* antimicrobial sensitivity testing including piperacillin (PIP), piperacillin-tazobactam (TZP), amikacin (AMK), ceftazidime (CAZ), aztreonam (ATM), imipenem (IPM), colistin (CST), gentamicin (GEN), and ciprofloxacin (CIP). The minimum inhibitory concentration (MIC) for each antimicrobial was determined by the double-fold dilution of antimicrobials (0.25–512 μg/mL) in cation-adjusted Mueller–Hinton broth (CAMHB) (Becton, Dickinson and Company (BD), Baltimore, MD, USA) according to the standards and guidelines of CLSI 2021 [57]. The dilution was performed in sterile 96-well flat microplates (50 µL of 2X antimicrobial/well). Then, 50 µL from fresh cultures (equivalent to 0.5 McFarland turbidity) were transferred to the individual wells of microplates. MIC was calculated as the lowest antimicrobial concentration that inhibits bacterial growth after aerobic incubation at 35 °C for 20 h. MDR was considered when isolates were resistant to three or more different antimicrobial classes [58]. The multiple antibiotic resistance (MAR) index was calculated for all isolates using the methodology of Krumperman et al. [59]. MIC50 and MIC90 were calculated according to Schwarz et al. [60].

### 2.7. Phenotypic Detection of ESBLs and MBLs

The double-disk synergy test (DDST) previously described by Jarlier et al. [61] was used for the phenotypic detection of ESBLs. A 0.5 McFarland standard suspension was prepared from the fresh inoculum of ceftazidime resistance (MIC > 8 mg/L) *P. aeruginosa* isolates then spread over the surface of Mueller–Hinton agar (Oxoid, Basingstoke, UK) plate. Discs of cefepime, cefotaxime, and aztreonam (30 μg each) were placed on Mueller–Hinton agar plates at a distance of 20 mm (centre to centre) from a disc containing amoxicillin/clavulanate (20/10 μg). The plates were then incubated at 37 °C for 18 h. ESBL production was considered if the zone diameter of the β-lactams was enlarged in the presence of clavulanate. The isolates that showed resistance to imipenem (MIC > 8 mg/L) were screened for MBL activity according to the methods of Giakkoupi et al. [62].

### 2.8. Detection of Selected Resistance Genes

The QIAGEN Plasmid Kits (Qiagen SA, Courtaboeuf, France) was used for the isolation and purification of the plasmid DNA according to the manufacturer’s instructions. The plasmid DNA was amplified for the detection of the *bla_TEM_*, *bla_SHV_*, *bla_CTX-M_*, *bla_IPM_*, and *bla_VIM_* genes according to Hosu et al. [55] in the LightCycler 2.0 instrument (Roche Applied Science, Penzberg, Germany). A total volume of 20 µL, containing 10 µL oasis PLUS 2X qPCR Master Mix (Primerdesign Ltd. Camberley, UK), two μL of primers (0.5 mM for each forward and reverse), two μL of the probe, and 50 ng of the DNA template was subjected to 95 °C for 10 min as an initial denaturation, followed by 40 cycles of denaturation at 95 °C for 15 s, and an annealing step at 60 °C for 30 s. Primers and protocols previously described were used for the sequencing of *bla_CTX-M_* [63,64]. All primer sequences are presented in Supplementary Materials Table S1.

### 2.9. Biofilm Formation

Bacterial biofilm production was determined by the microtiter plate method previously described by Stepanovic et al. [65]. Bacterial isolates were inoculated in BHI broth supplemented with sucrose (50 g/L) and then incubated overnight at 37 °C. The culture density from each isolate was adjusted to be approximately equivalent to 0.5 McFarland standard; 200 µL of the bacterial suspensions was transferred sterile 96-well polystyrene microtiter plates (Sigma-Aldrich, Saint Louis, MO, USA). A sterile BHI broth without bacteria and *P. aeruginosa* strain PA01 were used as the test controls. After incubation at 37 °C for 48 h, the plates were washed with sterile phosphate-buffered solution, left to air-dry, then stained with 2% crystal violet for 30 min. The plates were washed with sterile deionised water and air-dried, then 150 µL of absolute ethanol was added to each well. The optical density (OD) was measured at 570 nm in a plate reader (BioTek-800 ST, Shoreline, WA, USA). The experiment was performed in triplicate on three different days. Isolates were categorised into four groups (negative, weak, moderate, and strong biofilm producers) according to Stepanovic et al. [65].

### 2.10. Molecular Detection of Selected Virulence Factors and Quorum-Sensing (QS) Genes

Real-time PCR was performed to detect the following virulence genes: *lasB*, *toxA*, *lasR*, and *rhlR* according to the methods of Golpayegani et al. [66]. PCR was performed in a total volume of 20 µL, containing 10 µL oasig PLUS 2X qPCR Master Mix (Primerdesign Ltd. UK),1 µL (0.1 µM) of each primer and probe, and 50 ng of the DNA template. The thermal cycling was carried out in a LightCycler 2.0 instrument (Roche Applied Science, Penzberg, Germany) at 95 °C for 10 min as an initial denaturation, followed by 40 cycles of denaturation at 95 °C for 15 s, and an annealing step at 60 °C for 30 s.

### 2.11. Statistical Analysis

The Spearman’s rank correlation test and Fisher’s exact test or Chi-square test were used for the statistical analyses of the data (Prism 8 GraphPad Software, San Diego, CA, USA).

## 3. Results

### 3.1. Bacterial Isolation and Identification

A total of 180 uterine swabs and 90 water samples were processed for the presence of *P. aeruginosa*. Among the total samples, 54 *P. aeruginosa* isolates were recovered, with 17 isolates (31.5%) from camel, 15 isolates (27.8%) from cows, 12 isolates (22.2%) from mares, and 10 isolates (18.5%) from animal drinking water (Table 1).

All isolates were identified biochemically to the genus level by the automated Vitek 2 system with probability numbers >90%. Genetically, the *gyrB* gene was detected in all isolates and the sequences of the 16S rRNA gene showed >98% similarity to the existing *P. aeruginosa* database in GenBank. All sequences were deposited in the NCBI sequences database with GenBank accession numbers (OM943021–OM943074). There was no significant variation in the isolation frequencies across animal species or water samples (*p* > 0.05).

### 3.2. Molecular Typing

**Figure 1:**
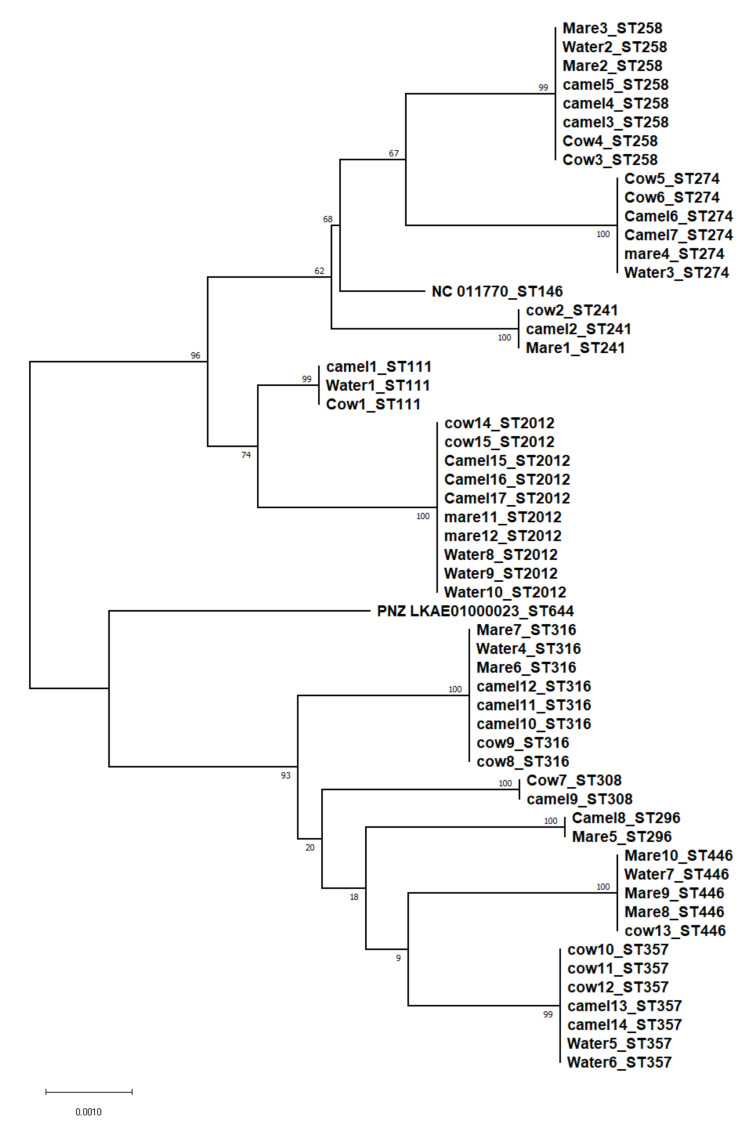
Unrooted phylogenetic trees using the concatenated sequences of the seven housekeeping genes of *P. aeruginosa* using the NJ method with the Kimura 2-parameter model for the distance calculations.

**Figure 2:**
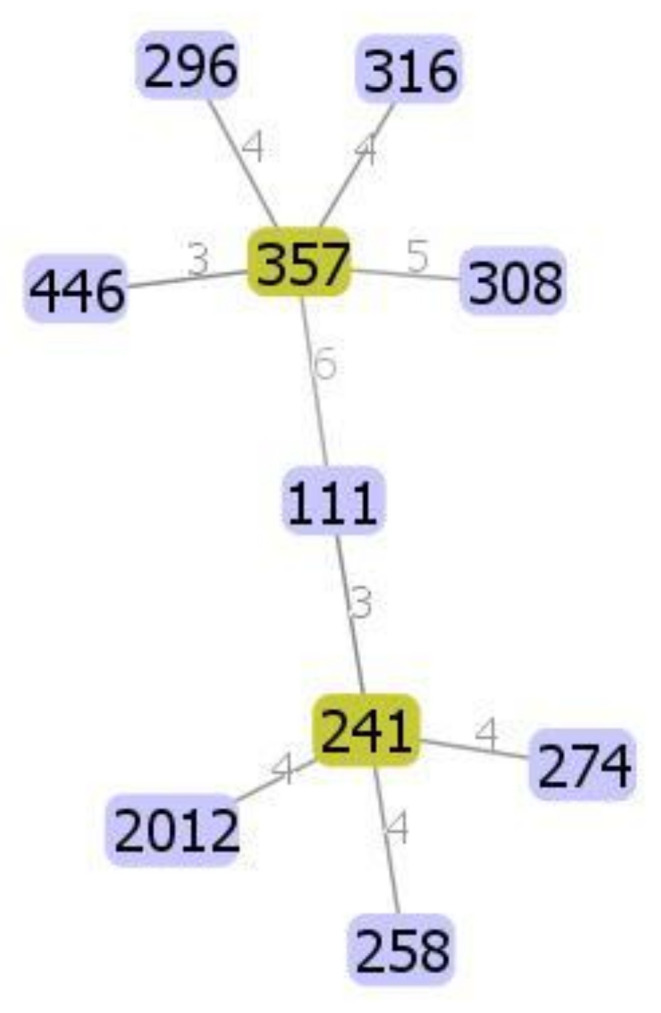
The goeBURST distance for the 10 sequence types obtained from the *P. aeruginosa* isolates (*n* = 54).

A total of ten different STs were assigned to the 54 *P. aeruginosa* isolates. The allelic profiles and STs are shown in Table 2 regarding the isolation source. The identification of ST2012 was significantly (*p* ≤ 0.05) higher than that of ST296, ST308, ST111, and ST241. The most commonly identified sequence type was ST2012 (10 isolates), followed by ST258, ST316 (eight isolates), ST357 (seven isolates), and ST274 (six isolates). ST296 and ST446 were not identified among the cow and camel isolates, respectively. On the other hand, three sequence types (ST111, ST308, and ST357) were not identified among the mare isolates. ST111, ST258, and ST2012 were identified in isolates recovered from camels and their drinking water while ST274 and ST357 were identified among the isolates recovered from cows and their drinking water. Three sequence types (ST316, ST446, and ST2012) were identified in isolates recovered from mares and their drinking water. ST2012 was identified in the drinking water of both camels and mares. To clarify the phylogenetic relationship between the isolates, the concatenated sequences of the seven housekeeping genes were used to construct a phylogenetic tree. The neighbour-joining (NJ) method was used to construct the tree with the distance calculated by the Kimura 2-parameter model. Fifty-six sequences were used including all isolates and two reference strains (*P. aeruginosa* NC 011660 and PNZ LKAE 0100023), as shown in Figure 1. eBURST analysis (https://pubmlst.org/organisms/pseudomonas-aeruginosa) (accessed on 10 January 2022) revealed that all sequence types were singletons. The goeBURST distance for the 10 ST constructed by PHYLOViZ version 2.0 is illustrated in Figure 2.

### 3.3. Antimicrobial Susceptibility

The results presented in Table 3 show the antimicrobial resistance of the 54 *P. aeruginosa* from different animal species and water. Overall, the highest resistance was against piperacillin (77.8%), ciprofloxacin (59.3%), gentamicin (50.0%), and ceftazidime (38.9%). Eight (14.8%) isolates showed resistance to imipenem, however, none of the isolates showed resistance to colistin. The number of resistant *P. aeruginosa* isolates from cow, camel, mare, and their drinking water are presented in Table 3. The MIC range, MIC50 and MIC90 for each antimicrobial are shown in Supplementary Materials Table S2.

**Figure 3:**
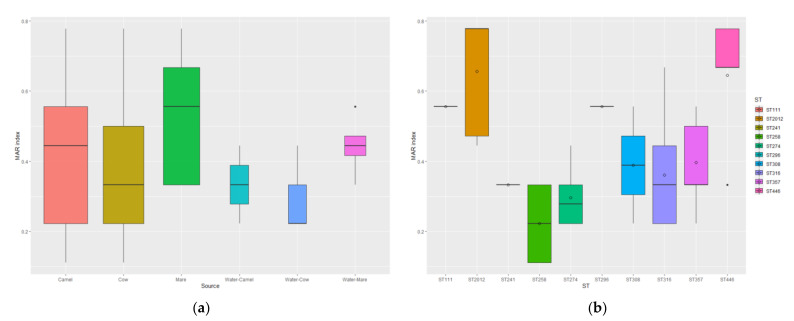
The mean MAR index values for the *P. aeruginosa* isolates (*n* = 54) regarding the source of isolation (**a**) and sequence types (**b**).

All *P. aeruginosa* isolates exhibited resistance to at least one of the tested antimicrobials. Table 4 shows the resistance profiles of the 54 *P. aeruginosa* isolates from different animal species. Three isolates (5.5%) were resistant to four antimicrobials, 10 (18.51%) showed resistance to five antimicrobials, three (5.5%) were resistant to six antimicrobials, and eight (14.81%) to seven antimicrobials. The MDR was observed in 24 isolates (44.44%) with a MAR index ranging from 0.44 to 0.77. No significant pairwise correlation (*p* = 0.33) was detected for STs versus the MAR index. Figure 3 shows the mean MAR index values for the *P. aeruginosa* isolates regarding the source of isolation and sequence types.

### 3.4. Phenotypic and Genotypic Detection of ESBLs and MBLs

ESBL-type enzyme production was detected in 21 out of 54 isolates (38.9%) in the DDST phenotypic assays. All ESBL producers were MDR with a MAR index ranging from 0.44 to 0.7. MBL production was found in five (62.5%) out of eight imipenem-resistant isolates. Of the 54 *P. aeruginosa* isolates, 21 were screened by real-time PCR for the detection of ESBL and MBL encoding genes. The results revealed that ESBL-genotypic resistant strains were positive for *bla*_TEM_ (90.47%), *bla_CTX-M_* (66.6%), and *bla*_SHV_ (42.8%). MBL-genotypic resistance, *bla_IPM_*, was not detected in all tested isolates while only the *bla_VIM_* was detected in seven isolates. Eight isolates showed the co-existence of ESBL and MBL genes. The different ESBL genotype combinations among the *P. aeruginosa* are presented in Table 5. Sequence analysis of the 12 *bla_CTX-M_* identified the CTX-M group1 β-lactamase; eight and six of them showed >98% similarity to the existing CTX-M15 and CTX-M1 database in GenBank, respectively. Sequences were deposited in the NCBI sequences database with GenBank accession numbers (ON185569–ON185574).

### 3.5. Biofilm Formation

**Figure 4:**
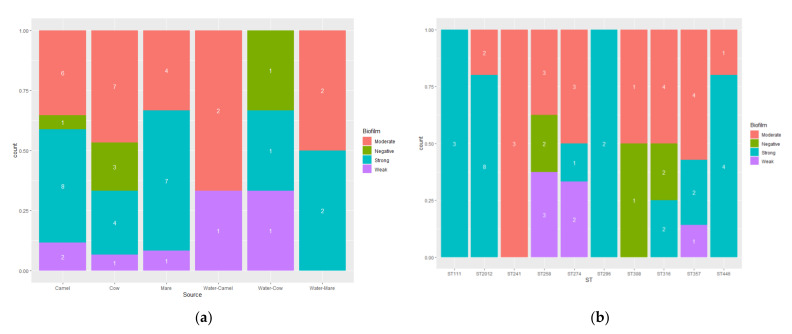
The frequency of the biofilm strength (negative, weak, moderate and strong) among the *P. aeruginosa* isolates (*n* = 54) with regard to the isolation source (**a**) and the isolate sequence type (**b**).

**Figure 5:**
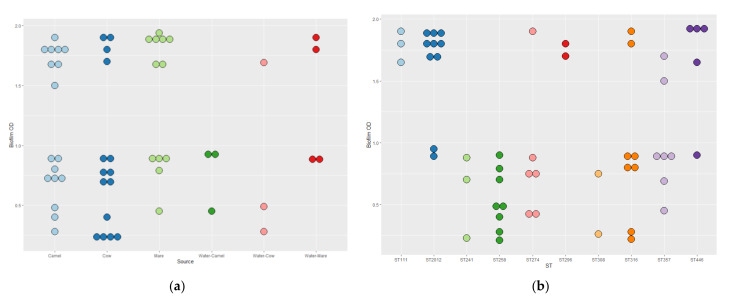
OD_570_ values indicate the amount of bacterial biofilm among the *P. aeruginosa* isolates (*n* = 54) with regard to the isolation source (**a**) and the isolate sequence type (**b**): negative (0.21–0.28), weak biofilm producers (0.4–0.49), moderate biofilm producers (0.7–0.95), and strong biofilm producers (1.5–1.94).

Biofilm phenotypes accounted for 90.7% (49/54) and were classified as follows: 11.1% (6/54) weak biofilm producers; 38.9% (21/54) moderate biofilm producers; 40.7% (22/54) strong biofilm producers. Figure 4 shows the distribution of the *P. aeruginosa* biofilm categories regarding the source of isolation and the sequence types. A significant pairwise correlation (*p* < 0.001) was determined for the MAR index versus biofilm formation (r = 0.731). No significant differences were found in the biofilm-forming OD in relation to the source of samples or the sequence types of the isolates (Figure 5).

### 3.6. Molecular Detection of Selected Virulence Factors and Quorum-Sensing (QS) Genes

The results of the real-time PCR revealed the amplification of virulence genes; the elastase (lasB) gene, exotoxin A (toxA) gene, and the QS genes (lasR and rhlR genes) in all *P. aeruginosa* isolates.

## 4. Discussion

In a global context, the “One Health” concept integrates molecular epidemiological aspects that add to the understanding of the evolution or genetic-relatedness of antimicrobial resistance in pathogens, the host (human/animal), and the associated ecosystem on a global scale.

In this work, *P. aeruginosa* isolates were detected in 44 of the 180 uterine swab samples (24.4%) from animals with endometritis, which is consistent with the results of several other previous studies [12,15,18,67]. *P. aeruginosa* is commonly recognised as a cause of endometritis in animals and is regarded as a venereal-transmitted pathogen [68,69].

Ten *P. aeruginosa* isolates were recovered from the drinking water of the animals. The drinking water of livestock could be contaminated with various bacteria including *P. aeruginosa*, which could infect large numbers of animals during a relatively brief period [41,70,71,72].

MLST represents an outstanding tool for global and long-term epidemiological studies. In this work, the ten STs were classified as singleton STs and no clonal complex (CC) could be obtained after eBURST analysis. This may suggest a high genetic diversity of *P. aeruginosa* isolated from the uterine infection of animals. Seven STs were identified from both animals and their drinking water, which may indicate that animals with clinical uterine infection are the source of water contamination at the farm.

Among the 10 STs reported in this work, five STs (ST111, ST274, ST308, ST357, and ST446) were reported as *P. aeruginosa* high risk clones [73]. These STs are founders for the clonal complexes CC111, CC274, CC308, CC357, and CC446 [73].

The most common STs in the current work (ST2012, ST258, ST357, and ST274) have previously been identified in Saudi Arabia and the Gulf region [74,75]. The identified STs in this study are internationally widespread clones associated with human outbreaks and sometimes with multidrug resistance phenotypes [76,77,78,79]. However, studies on the MLST of *P. aeruginosa* from animals are scarce.

For the treatment of metritis, antibiotics are given by the intrauterine route, systemically, or both [80,81]. Each use of an antimicrobial drug is inherently associated with the selective pressure on the resistant bacterial population, which stresses the importance of their prudent use [82,83].

In this work, a variable resistance to β-lactams was observed among the *P. aeruginosa* isolates. A high proportion of resistance to piperacillin (77.8%), and piperacillin/tazobactam (59%) was observed among the *P. aeruginosa* isolates, which is consistent with previous studies [84,85,86]. *P. aeruginosa* possesses a naturally-occurring AmpC β-lactamase that is not inhibited by the currently available lactam inhibitors, clavulanic acid, sulbactam, and tazobactam, and therefore confers resistance to antibiotic combinations [87,88]. Several earlier studies have reported high proportions of *P. aeruginosa* resistance to ceftazidime and aztreonam [84,89,90,91]. This is consistent with the 39% resistance observed in this study.

Carbapenems are critically-important antibiotics, restricted to human use [92]. In this work, 14.8% (8/54) of the isolates showed a resistance to imipenem; this is inconsistent with the results of previous studies [93,94,95]. The occurrence of carbapenem-resistant bacteria was reported in veterinary medicine [84,90,96,97,98]. Moreover, the circulation of carbapenem-resistant bacteria between humans, animals, and the environment was also reported [99,100,101,102].

Acquired β-lactamases in *P. aeruginosa* have recently been reported including the ESBL enzymes that are able to hydrolyse a wider range of β-lactams [103]. In this study, the most prevalent β-lactamase encoding genes were *bla_TEM_*, *bla_SHV_*, *bla_CTX-M_*; this is inconsistent with previous reports [55,104,105]. The *bla_CTX-M_* genes were classified as CTX-M group I with a 98% similarity with the CTX-M-15 and CTX-M-1 genes. The CTX-M group 1 variants (CTX-M-1 and CTX-M-15) have previously been identified in Saudi Arabia [106,107]. CTX-M group I is responsible for hydrolysing the broad-spectrum cephalosporins and aztreonam, and also confers resistance to penicillins [103,108]. The *bla_IPM_* gene was not detected by PCR in this study, whereas the *bla_VIM_* gene was identified in seven isolates, inconsistent with previous studies [49,109,110,111,112] and in contrast to Ejikeugwu et al. [113], who identified only *bla_IPM_* among their isolates. These discrepancies may be due to the variations in the geographic locations, antibiotic use, and antibiotic stewardship practices.

MDR was frequently detected among the isolated *P. aeruginosa* in this study. The emergence of MDR *P. aeruginosa* has also been reported worldwide [114,115,116].

In the absence of new anti-pseudomonal drugs, clinicians have had to resort to older antimicrobials such as colistin for the treatment of MDR resistant isolates [117,118]. In this work, none of the isolates showed a resistance to colistin. Colistin-resistant *P. aeruginosa* is uncommon and is rarely MDR [119]. However, nephrotoxicity and neurotoxicity are the most-common potential toxicities with the parenteral administration of colistin and is mainly used as salvage therapy in the treatment of often life-threatening infections due to the MDR of *P. aeruginosa* [117,119].

One of the most important features of microbial biofilms is that the bacteria are able to survive antibiotic treatments administered at high doses [120]. In this study, biofilm formation was observed in 90.7% of the isolates; this is concordant with previous studies [121,122]. A significant positive correlation was observed between biofilm production and both the MAR index and MDR. Biofilm production has been linked to antibiotic resistance in *P. aeruginosa* [123].

*P. aeruginosa* uses several pathogenicity factors to interfere with the host’s defences [5,124]. In the current work, the *lasB*, *toxA*, *lasR*, and *rhlR* genes were detected in all *P. aeruginosa* isolates, which is inconsistent with [125,126]. In contrast, a lower detection rate of the virulence genes was reported by Osman et al. [127]. Variations in the virulence genes were observed in the clinical *P. aeruginosa* isolates from different geographic areas [128,129,130].

A limitation of this study was the lack of complete information on the antimicrobial use on farms due to the absence of records at these small farms. However, the antimicrobials used at these farms are not used in a judicious way, the farmer can obtain antimicrobials without a veterinary prescription, and oxytetracycline was the most widely used antimicrobial on these farms. Further investigation of the risk factors associated with the spread of MDR of *P. aeruginosa* in animals and the environment is highly recommended.

## 5. Conclusions

This study concluded that *P. aeruginosa* is one of the most common bacteria associated with endometritis in cows, camels, and mares in the Eastern Region, Saudi Arabia. Moreover, our results provide further evidence on the emergence of MDR and carbapenem-resistant *P. aeruginosa.* This is the first study to investigate the sequence types of *P. aeruginosa* from animals in Saudi Arabia. The results revealed the emergence of the *P. aeruginosa* high-risk clones ST111, ST274, ST308, ST357, and ST446 in animals with clinical uterine inflammation and their drinking water. Molecular identification of *bla_TEM_*, *bla_SHV_*, *bla_CTX-M_,* and *bla_VIM_* genes in the plasmid DNA emphasises the horizontal transmission of antimicrobial-resistance genes. Furthermore, proactive antimicrobial agent control measures should be developed to limit the spread of multidrug-resistant strains. Further studies are needed for the investigation of the molecular mechanism.

## Figures and Tables

**Table 1:** Percentage (N/N) of *P. aeruginosa* isolates recovered from the uterine swabs of different animal species and the drinking water.

Sample Type	Cow	Camel	Mare	Total
Uterine swabs	21.4 (15/70)	28.3 (17/60)	24 (12/50)	24.4 (44/180)
Drinking water	10 (3/30)	10 (3/30)	13.3 (4/30)	11.1 (10/90)

**Table 2:** The number of strains for each sequence type for *P. aeruginosa* isolates (*n* = 54).

Sequence Type (ST)	N (%)	Uterine Swab			Drinking Water		
		**Cow**	**Camel**	**Mare**	**Cow**	**Camel**	**Mare**
111	3 (5.56) ^bc^	1	1	0	0	1	0
241	3 (5.56) ^bc^	1	1	1	0	0	0
258	8 (14.82) ^ab^	2	3	2	0	1	0
274	6 (11.11) ^abc^	2	2	1	1	0	0
296	2 (3.70) ^c^	0	1	1	0	0	0
308	2 (3.70) ^c^	1	1	0	0	0	0
316	8 (14.82) ^ab^	2	3	2	0	0	1
357	7 (12.96) ^abc^	3	2	0	1	0	0
446	5 (9.26) ^abc^	1	0	3	0	0	1
2012	10 (18.52) ^a^	2	3	2	0	1	2

^a,b,c^ N (%) in a column without a common superscript letter differs (*p* ≤ 0.05).

**Table 3:** The antimicrobial-resistant profiles of *P. aeruginosa* (*n* = 54) isolated from cow, camel, mare, and drinking water.

Antimicrobials	Breakpoints	N (%)	No. of Resistant *P. aeruginosa* Isolates (%)					
			Uterine Swab			Drinking Water		
			Cow	Camel	Mare	Cow	Camel	Mare
Piperacillin	S ≤ 16 R ≥ 128	42 (77.78) ^a^	11 (26.1)	15 (35.7)	10 (23.8)	3 (7.1)	1 (2.3)	2 (4.7)
Piperacillin/Tazobactam	S ≤ 16 R ≥ 128	32 (59.26) ^b^	6 (18.7)	10 (31.2)	10 (31.2)	1 (3.1)	2 (6.2)	3 (9.3)
Ceftazidime	S ≤ 8 R ≥ 32	21 (38.89) ^c^	6 (28.5)	6 (28.5)	7 (33.3)	1 (4.7)	0 (0)	1 (4.7)
Aztreonam	S ≤ 8 R ≥ 32	21 (38.89) ^c^	6 (28.5)	6 (28.5)	7 (33.3)	1 (4.7)	0 (0)	1 (4.7)
Imipenem	S ≤ 2 R ≥ 8	8 (14.82) ^d^	2 (25)	3 (37.5)	3 (37.5)	0 (0)	0 (0)	0 (0)
Amikacin	S ≤ 16 R ≥ 64	27 (50) ^bc^	8 (29.6)	9 (33.3)	4 (14.8)	2 (7.4)	1 (3.7)	3 (11.1)
Gentamicin	S ≤ 4 R ≥ 16	27 (50) ^bc^	8 (29.6)	9 (33.3)	4 (14.8)	2 (7.4)	1 (3.7)	3 (11.1)
Ciprofloxacin	S ≤ 0.5 R ≥ 2	32 (59.25) ^b^	10 (31.2)	10 (31.2)	10 (31.2)	1 (3.1)	1 (3.1)	0 (0)
Colistin	S ≤ 0.001 R ≥ 4	0 (0) ^e^	0 (0)	0 (0)	0 (0)	0 (0)	0 (0)	0 (0)

^a–e^ Values in a column without a common superscript letter differ (*p* ≤ 0.05).

**Table 4:** Antimicrobial-resistance profile of *P. aeruginosa* isolates (*n* = 54) recovered from cow, camel, mare, and drinking water.

Resistance Pattern	MDR	No.	Uterine Swab			Drinking Water		
			Cow	Camel	Mare	Cow	Camel	Mare
CIP		1	1	0	0	0	0	0
GEN AMK		3	0	0	2	0	0	1
GEN CIP AMK		7	3	2	0	1	1	0
PIP		2	0	2	0	0	0	0
PIP CIP		6	3	3	0	0	0	0
PIP GEN AMK		2	2	0	0	0	0	0
PIP TZP		2	0	0	0	1	0	1
PIP TZP CIP		3	0	0	3	0	0	0
PIP TZP GEN AMK		4	0	2	0	0	0	2
PIP TZP GEN CIP AMK	MDR	2	0	2	0	0	0	0
PIP TZP CAZ ATM	MDR	3	1	0	0	1	1	0
PIP TZP CAZ ATM CIP	MDR	3	0	0	3	0	0	0
PIP TZP CAZ ATM GEN CIP AMK	MDR	8	3	3	2	0	0	0
PIP TZP CAZ ATM IPM	MDR	5	2	3	0	0	0	0
PIP TZP CAZ ATM IPM CIP	MDR	3	0	0	2	0	1	0

Ciprofloxacin (CIP); amikacin (AMK); piperacillin (PIP) piperacillin/tazobactam (TZP); aztronam (ATM); gentamicin (GEN); ceftazidime (CAZ); imipenem (IPM).

**Table 5:** Beta-lactamase gene types in phenotypic ESBL and MBL *P. aeruginosa* (*n* = 21) recovered from uterine swabs and drinking water.

Profile	No.	Uterine Swab			Drinking Water		
		Cow	Camel	Mare	Cow	Camel	Mare
*bla* TEM	4	1	2	1	0	0	0
*bla* SHV	1	0	0	1	0	0	0
*bla* SHV-*bla*CTX-M	1	1	0	0	0	0	0
*bla* TEM-*bla*CTX-M	3	1	0	2	0	0	0
*bla* TEM-*bla*CTX M-*bla*VIM	4	0	2	2	0	0	0
*bla* TEM-*bla*SHV-*bla*CTX-M	4	1	1	1	0	0	1
*bla* TEM-*bla*SHV-*bla*VIM	3	2	0	1	0	0	0
*bla* TEM-*bla*VIM	1	0	1	0	0	0	0

## Data Availability

The data presented in this study are available on request from the corresponding author.

## Supplementary Materials

The following supporting information can be downloaded at: https://www.mdpi.com/article/10.3390/vetsci9050239/s1, Table S1: Antimicrobial resistance, MIC50, MIC90, MIC range for 54 *P*. *aeruginosa* isolates recovered from cow, camel, mare, and their drinking water; Table S2: Primer and probes used for amplification of different genes in *P. aeruginosa* isolates.
