# Supplementary material for: Genetic Diversity, Biofilm Formation, and Antibiotic Resistance of Pseudomonas aeruginosa Isolated from Cow, Camel, and Mare with Clinical Endometritis
Source: Vet Sci. 2022 May 16;9(5):239. doi: 10.3390/vetsci9050239 (PMC9147788; doi:10.3390/vetsci9050239)
Supplement: Supplementary file 1 [file vetsci-09-00239-s001.zip › vetsci-1667377-supplementary.pdf]

Table S1: Antimicrobial resistance, MIC50, MIC90, MIC range for 54 *P. aeruginosa* isolates recovered from cow, camel, mare, and their drinking water.

Antibiotic name	Breakpoints	Isolates	Number	%R	%R 95%C.I.	MIC50	MIC90	Geom.Mean	MIC Range	<=.001	0.016	0.032	0.064	0.125	0.25	0.5	1	2	4	8	16	32	64	128	256	512
Piperacillin	S<=16 R>=128	All isolates	54	77.8	64.1-87.5	128	512	120.043	8 - 512											5	7			17	14	11
		cow isolates	15	73.3	44.8-91.1	128	512	97.006	8 - 512											2	2			6	2	3
		camel isolates	17	88.2	62.2-97.9	256	512	177.366	16 - 512												2			5	6	4
		mare isolates	12	83.3	50.8-97.0	128	512	143.675	8 - 512											1	1			4	3	3
		water isolates	10	60	27.4-86.3	128	256	68.593	8 - 512											2	2			2	3	1
Piperacillin/Tazobactam	S<=16 R>=128	All isolates	54	59.3	45.1-72.2	128	256	50.797	2 - 512									1	1	12	8			22	9	1
		cow isolates	15	40	17.5-67.1	8	256	25.398	2 - 256									1		7	1			4	2	
		camel isolates	17	58.8	33.4-80.6	128	256	54.369	8 - 512											3	4			8	1	1
		mare isolates	12	83.3	50.8-97.0	128	256	101.594	4 - 256										1		1			6	4	
		water isolates	10	60	27.4-86.3	128	256	55.715	8 - 256											2	2			4	2	
Ceftazidime	S<=8 R>=32	All isolates	54	38.9	26.2-53.1	8	64	15.466	2 - 164									2	8	23		3	15	3		
		cow isolates	15	40	17.5-67.1	8	64	13.3	2 - 64									2	1	6		2	4			
		camel isolates	17	35.3	15.3-61.4	8	128	16.911	4 - 164										2	9			4	2		
		mare isolates	12	58.3	28.6-83.5	32	64	25.398	4 - 128										1	4		1	5	1		
		water isolates	10	20	3.5-55.8	8	64	9.19	4 - 64										4	4			2			
Aztreonam	S<=8 R>=32	All isolates	54	38.9	26.2-53.1	8	64	14.692	2 - 164									1	9	23		7	12	2		
		cow isolates	15	40	17.5-67.1	8	64	12.126	2 - 64									1	4	4		3	3			
		camel isolates	17	35.3	15.3-61.4	8	64	14.964	4 - 164										2	9		2	3	1		
		mare isolates	12	58.3	28.6-83.5	32	64	23.973	4 - 128										1	4		2	4	1		
		water isolates	10	20	3.5-55.8	8	64	10.556	4 - 64										2	6			2			
Imipenem	S<=2 R>=8	All isolates	54	14.8	7.0-27.7	1	8	1.293	0.125 - 8					1		8	30	7		8						
		cow isolates	15	13.3	2.3-41.6	1	8	1.203	0.125 - 8					1		2	7	3		2						
		camel isolates	17	17.6	4.6-44.1	1	8	1.566	0.5 - 8							2	8	4		3						
		mare isolates	12	25	6.7-57.2	1	8	1.587	0.5 - 8							1	8			3						
		water isolates	10	0	0.0-34.5	1	1	0.812	0.5 - 1							3	7									
Amikacin	S<=16 R>=64	All isolates	54	50	36.3-63.7	16	128	30.011	2 - 256									1	3	15	8		10	15	2	
		cow isolates	15	53.3	27.4-77.7	64	256	27.858	2 - 256									1	2	4			3	3	2	
		camel isolates	17	52.9	28.5-76.1	64	128	34.719	8 - 128											4	4		4	5		
		mare isolates	12	33.3	11.3-64.5	16	128	22.627	4 - 128										1	3	4		1	3		
		water isolates	10	60	27.4-86.3	64	128	36.758	8 - 128											4			2	4		
Gentamicin	S<=4 R>=16	All isolates	54	50	36.3-63.7	4	32	8.752	2 - 64									21	6		4	18	5			
		cow isolates	15	53.3	27.4-77.7	16	64	10.079	2 - 64									5	2		1	5	2			
		camel isolates	17	52.9	28.5-76.1	16	32	9.417	2 - 64									6	2		1	7	1			
		mare isolates	12	33.3	11.3-64.5	2	32	5.657	2 - 64									6	2		1	2	1			
		water isolates	10	60	27.4-86.3	16	32	10.556	2 - 64									4			1	4	1			
Ciprofloxacin	S<=.5 R>=2	All isolates	54	59.3	45.1-72.2	2	8	1.414	0.125 - 16					8	3	11		8	13	10	1					
		cow isolates	15	66.7	38.7-87.0	4	8	2	0.125 - 16					2		3		2	3	4	1					
		camel isolates	17	58.8	33.4-80.6	4	8	1.386	0.125 - 8					4		3		1	5	4						
		mare isolates	12	83.3	50.8-97.0	2	8	2.378	0.25 - 8						1	1		4	4	2						
		water isolates	10	20	3.5-55.8	0.5	2	0.467	0.125 - 4					2	2	4		1	1							
Colistin	S<=.001 R>=4	All isolates	54	0	0.0-8.3	0.001	0.001	0.001	0.001 - 0.001	54																
		cow isolates	15	0	0.0-25.3	0.001	0.001	0.001	0.001 - 0.001	15																
		camel isolates	17	0	0.0-22.9	0.001	0.001	0.001	0.001 - 0.001	17																
		mare isolates	12	0	0.0-30.1	0.001	0.001	0.001	0.001 - 0.001	12																
		water isolates	10	0	0.0-34.5	0.001	0.001	0.001	0.001 - 0.001	10																

Table S2: Primer and probes used for amplification of different genes in P. aeruginosa isolates.

Genes	Oligo	Oligonucleotide sequences (5′-3′)	References
lasB	lasB-F	CATGTCGGAGAACGCTTCG	[66]
	lasB-R	GGCTTCACCGAGCAGAACTC	
	lasB-P	FAM-CATTCCGCCTGATTGCCCGC-BHQ1	
toxA	toxA-F	ACCCGGCGAAGCATGAC	
	toxA-R	GGGAAATGCAGGCGATGAC	
	toxA-P	HEX -TGGACATCAAGCCCACGGTCATCA -BHQ1	
lasR	lasR-F	CAGCCGGGAGAAGGAAGTG	
	lasR-R	TCCGAGCAGTTGCAGATAACC	
	lasR-P	FAM-TGCGCCATCGGCAAGACCAGT-BHQ1	
rhlR	rhlR-F	GCTGCGTTGCATGATCGAG	
	rhlR-R	CAGACCGGGTTGGACATCAG	
	rhlR-P	HEX- TGCTGACCCAGAAGCTGACCGACC BHQ1	
gyrB	gyrB-F	CCT GAC CAT CCG TCG CCA CAA	[55]
	gyrB-R	CGC AGC AGG ATG CCG ACG CC	
	gyrB-P	FAM-CCG TGG TGG TAG ACC TGT TCC CAG ACC-BHQ	
CTX-M	CTX-M-F	ATGAGYACCAGTAARGTKATGGC	
	CTX-M-R	ATCACKCGGRTCGCCIGGRAT	
	CTX-M-P	FAM-CCCGACAGCTGGGAGACGAAACGT-BBQ	
SHV	SHV-F	TCCCATGATGAGCACCTTTAAA	
	SHV-R	TCCTGCTGGCGATAGTGGAT	
	SHV-P	FAM-TGCCGGTGACGAACAGCTGGAG-BBQ	
TEM	TEM-F	GCATCTTACGGATGGCATGA	
	TEM-R	GTCCTCCGATCGTTGTCAGAA	
	TEM-P	FAM-CAGTGCTGCCATAACCATGAGTGA-BHQ1	
IMP	IMP-F	GGGCGGAATAGAGTGGCTTA	
	IMP-R	GGCTTGAACCTTACCGTCTTTTT	
	IMP-P	FAM-CGATCTATCCCCACGTATGCATCTGAATTAACA-BHQ1	
VIM	VIM-F	TGCGCTTCGGTCCAGTAGA	
	VIM-R	TGACGGGACGTATACAACCAGAT	
	VIM-P	FAM-CTTCTATCCTGGTGCTGCGCATTCG-BHQ1	
16S rRNA	27F	AGAGTTTGATCCTGGCTCAG	[56]
	1492R	TACGGYTACCTTGTTACGACTT	
CTX-M group I	CTXM1-F3	GAC GAT GTC ACT GGC TGA GC	[63]
	CTXM1-R2	AGC CG C CGA CGC TAA TAC A	
CTX-M group I	TOHO1-2F	GCG ACC TGG TTA ACT ACA ATC C	
	TOHO1-1R	CGG TAG TAT TGC CCT TAA GCC	
CTX-M group I	CTXM825F	CGC TTT GCC ATG TGC AGC ACC	
	CTXM825R	GCT CAG TAC GAT CGA GCC	
CTX-M group I	CTXM914F	GCT GGA GAA AAG CAG CGG AG	
	CTXM914R	GTA AGC TGA CGC AAC GTC TG	

Reference

55. Hosu, M.C.; Vasaikar, S.D.; Okuthe, G.E.; Apalata, T.J.Detection of extended spectrum beta-lactamase genes in Pseudomonas aeruginosa isolated from patients in rural Eastern Cape Province, South Africa. Sci. Rep. 2021, 11, 7110.
56. Weisburg, W.G.; Barns, S.M.; Pelletier, D.A.; Lane, D.J. 16S ribosomal DNA amplification for phylogenetic study. J. Bacteriol. 1991, 173, 697 – 703. https://doi.org/10.1128/jb.173.2.697-703.1991.
63. Pitout, J.D.; Thomson, K.S.; Hanson, N.D.; Ehrhardt, A.F.; Moland, E.S.; Sanders, C.C. beta-Lactamases responsible for resistance to expanded-spectrum cephalosporins in Klebsiella pneumoniae, Escherichia coli, and Proteus mirabilis isolates recovered in South Africa. Antimicrob. Agents Chemother. 1998, 42, 1350 – 1354. https://doi.org/10.1128/aac.42.6.1350.
66. Golpayegani, A.; Nodehi, R.N.; Rezaei, F.; Alimohammadi, M.; Douraghi, M.J. Real-time polymerase chain reaction assays for rapid detection and virulence evaluation of the environmental Pseudomonas aeruginosa isolates. Mol. Biol. Rep. 2019, 46, 4049 – 4061.
